# The dual nature of the human face: there is a little Jekyll and a little Hyde in all of us

**DOI:** 10.3389/fpsyg.2014.00139

**Published:** 2014-03-06

**Authors:** Karolann Robinson, Caroline Blais, Justin Duncan, Hélène Forget, Daniel Fiset

**Affiliations:** ^1^Département de Psychoéducation et de Psychologie, Université du Québec en OutaouaisGatineau, QC, Canada; ^2^Centre de Recherche en Neuropsychologie et CognitionMontréal, QC, Canada

**Keywords:** visual information extraction, social face perception, social judgments, trust, dominance

## Abstract

The fact that a mere glance makes it possible to extract a wealth of information about the person being observed is testament to both the salience of the human face and the brain’s high efficiency in processing this information. Prior work has revealed that social judgments of faces are determined by facial features that vary on two orthogonal dimensions: trustworthiness and dominance. We conducted two experiments to investigate the visual information subtending trustworthiness and dominance judgments. In Experiment 1, we used the Bubbles technique to identify the facial areas and the spatial frequencies that modulate these two judgments. Our results show that the eye and mouth areas in high-to-medium spatial frequency bands were positively correlated with judgments of trustworthiness; the eyebrows region in medium-to-low frequency bands was positively correlated with judgments of dominance; and the lower left jawbone in medium-to-low frequency bands was negatively correlated with judgments of dominance. In Experiment 2, we used the results of Experiment 1 to induce subtle variations in the relative contrast of different facial areas, and showed that it is possible to rig social perception using such a manipulation.

## INTRODUCTION

The human face is an extremely powerful stimulus and its adequate perceptual analysis is critical in social relationships. From a mere glance, it is possible to extract a wealth of information about the gender, race, age, emotional state and identity of the person being observed (e.g., Bruce and Young, 1998). Relying solely on facial information, humans are able to form a first impression within a few milliseconds (Bar et al., 2006; Willis and Todorov, 2006). In fact, many studies have shown that people infer personality traits (Hassin and Trope, 2000; Bar et al., 2006; Little and Perrett, 2007; Rule et al., 2009; Walker and Vetter, 2009; Todorov et al., 2011) and make social judgments (Oosterhof and Todorov, 2008; Zebrowitz and Montepare, 2008; Todorov et al., 2013) based on the facial appearance of others.

 A high level of agreement is found among individuals’ judgments (Zebrowitz-McArthur and Berry, 1987; Albright et al., 1997; Zebrowitz and Montepare, 2008), suggesting that some of the visual information contained in a face correlates with social judgments (Oosterhof and Todorov, 2008; Walker and Vetter, 2009). Surprisingly, few authors have investigated the physical characteristics of faces underlying social judgments; Thus, in this respect, Oosterhof and Todorov (2008) stand as pioneers. They have identified a wide variety of features spontaneously used to categorize neutral faces, and then collected judgments on these dimensions. They found that the judgments were highly correlated with each other and that two orthogonal dimensions could account for the observed variance: trustworthiness and dominance. Based on this work, they created a two dimensional space in which each specific social judgment can be represented as a function of these two dimensions, and then built a statistical model to represent how faces vary along these dimensions, thereby showing that most of the changes occur on the facial features contained within the regions of the eyes, eyebrows and mouth, as well as in the face contour.

Extending these results, Dotsch and Todorov (2012) used reverse correlation (Ahumada and Lovell, 1971; Kontsevich and Tyler, 2004; Mangini and Biederman, 2004) to examine the representational content of trustworthiness and dominance judgments. In this specific version of the technique, each stimulus was created by adding an amount of visual noise to a base face that remained constant throughout the experiment. The participants were asked to decide which of two simultaneously presented stimuli (made up of the same base face, but processed with two opposite patterns of visual noise) was the best example of the evaluated dimension. From the participants’ responses, Dotsch and Todorov (2012) were able to generate classification images (CIs) of trustworthiness and dominance judgments, and of their opposites (untrustworthiness and submissiveness). They also performed a cluster test (Chauvin et al., 2005) on each CI to identify facial regions on which the emphasis was put in the mental representations of the aforementioned social judgments. The results showed that trustworthiness and untrustworthiness judgments are predicted by pixel luminance in the eye/eyebrows and the mouth areas. Alternatively, dominance and submissiveness judgments are driven by information in the eyebrows and jaw areas.

The reverse correlation technique used by Dotsch and Todorov (2012) involves alteration of the appearance of the features, and therefore allows to make inferences on the features’ shapes in the visual representation associated with a trustworthy or dominant face. Similarly, the model developed by Oosterhof and Todorov (2008) also involves a manipulation of the shape of the facial features. For instance, in the modelisation of face trustworthiness developed by Oosterhof and Todorov (2008), the eyebrows clearly convey anger at the negative end of the trustworthiness judgment. The same goes for the results obtained by Dotsch and Todorov (2012) using reverse correlation. Both of these studies were very informative with regards to how the appearance and the shape of different features is associated with the percept of trust and dominance.

In the present study, we explored the possibility of biasing trustworthiness and dominance judgments without altering the facial features’ shapes (Experiment 1), or altering them very subtly (Experiment 2). The present study is also aimed at furthering our understanding of the visual information underlying social judgments by investigating in which spatial frequencies the different facial areas are processed during trustworthiness and dominance judgments. We used the Bubbles technique (Gosselin and Schyns, 2001) to probe the facial features and the spatial frequencies that, when utilized by an observer, lead to changes in the perceived trustworthiness and dominance of a human face. We thought Bubbles best suited to our experiment since it does not alter the shape of the facial features; rather, it either reveals or hides them (for a discussion of the differences between reverse correlation and Bubbles, see Gosselin and Schyns, 2004). Our investigation also takes it one step further: in a novel use of the Bubbles’ results, we validate our findings by experimentally inducing a change in the perception of trustworthiness and dominance. In doing so, we reveal the dual nature of faces: that a person can be made to look more or less trustworthy/dominant by over- and under-representing the same features, respectively; that a person is, to some extent, both Dr. Jekyll and Mr. Hyde.

## EXPERIMENT 1

The Bubbles method was used to verify what visual information biases facial judgments toward higher (vs. lower) trustworthiness and higher (vs. lower) dominance. This technique allows to reveal the potent information (Gosselin and Schyns, 2002) for a task, i.e., the interaction between the represented (psychological construct of the observer) and the available information (i.e., physical information contained in the stimulus). It consists in applying Gaussian windows on a stimulus to reveal random subsets of visual information (see below for more details). This therefore does not modify the physical shape of the facial features.

### METHODS

#### Participants

A total of 50 participants were recruited for each part of the first experiment (43 partook in both parts, i.e., trustworthiness and dominance). All participants were Caucasian and had normal or corrected-to-normal visual acuity.

#### Material and stimuli

**FIGURE 1:**
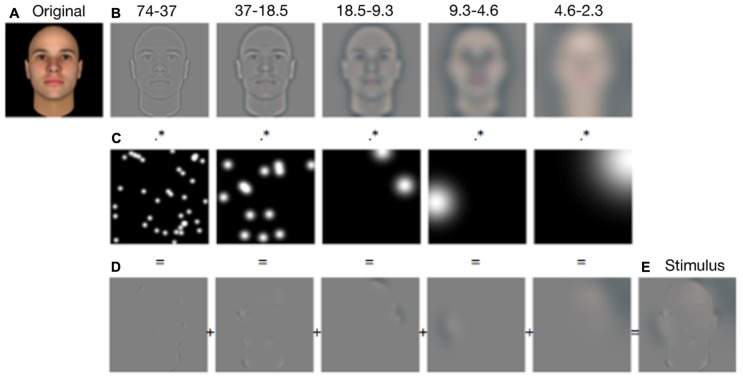
**Illustration of the stimulus-generation process for Experiment 1.** Each original stimulus **(A)** was first decomposed into five spatial-frequency bandwidths **(B)**. Each bandwidth was then independently sampled with randomly positioned Gaussian windows (i.e., bubbles), so that sparse information was revealed **(C)**. The information samples were summed across the five scales **(D)** to produce an experimental stimulus **(E)**.

The stimuli consisted in 300 computer-generated faces (Singular Inversions, 2005; Oosterhof and Todorov, 2008). We chose this database to have better control over non-facial features such as skin texture, hair, freckles, etc. All the pictures depicted a front-view and eyes-open Caucasian male face with a neutral expression. The pictures were spatially aligned on the positions of the main internal facial features (eyes, mouth, and nose) using translation, rotation, and scaling manipulations. The faces subtended 6.1° of visual angle on average. To create a Bubblized stimulus, the picture of a face (see **Figure 1A**) was first bandpass filtered into five non-overlapping spatial frequency bands (74–37, 37–18.5, 18.5–9.3, 9.3–4.6, 4.6–2.3 cycles per face; the remaining bandwidth served as a constant background; see **Figure 1B**), using the Pyramid toolbox for Matlab (Simoncelli, 1999). Second, each spatial frequency band was independently and randomly sampled using Gaussian apertures (or bubbles) of varying standard deviations; that is, the size of the bubbles was adjusted according to frequency band, revealing three cycles per band (see **Figure 1C**; standard deviations of the bubbles were 0.14, 0.28, 0.56, 1.12, and 2.24° of visual angle from the finest to the coarsest scale). Because the size of the bubbles increased as the spatial scale became coarser, the number of bubbles differed at each scale to maintain constant the probability of revealing a given pixel in each spatial frequency band. The total number of bubbles (i.e., 50 bubbles) was kept constant across trials and across participants. Finally, the five randomly sampled images plus the background were summed to produce the experimental stimulus (see **Figures 1D,E**). Note that the number of bubbles was determined based on the shared subjective impression of three of the authors (Karolann Robinson, Daniel Fiset, Justin Duncan) that 50 bubbles was a good compromise between percept modulation and face visibility. One of the objectives of Experiment 2 was to address the possibility that our results could have been an artifact of the arbitrary number of bubbles.

The stimuli were displayed on a 23-inch Samsung LED monitor set with a refresh rate of 120 Hz. The experiment ran on an Apple MacPro QuadCore computer. The experimental program was written in Matlab, and used functions of the Psychophysics Toolbox (Brainard, 1997; Pelli, 1997).

#### Procedure

Two tasks containing 600 trials each were conducted. In one of the tasks, the participants were asked to make trustworthiness judgments, and in the other, they were asked to make dominance judgments. The order in which the two tasks were conducted was counterbalanced for the 43 participants who took part in both. In each task, the first 300 trials consisted in the presentation of bubblized faces, and the next 300 trials consisted in the presentation of their unaltered counterparts. On each trial, the participants were asked to judge a face on its level of trustworthiness or dominance on a scale that ranged from 1 (*not trustworthy* or *not dominant* at all) to 9 (*very trustworthy* or *very dominant*). Participants were told to rely on their *gut feeling* and that there was no right or wrong answer. The faces were presented in random order. Judgments obtained for fully visible faces served as the baseline, while judgments obtained from the bubblized versions of the same faces were assumed to reflect the influence of the experimental manipulation on the baseline judgment; we named this influence the “Bubbles-Induced Bias” (BIB). Each trial went as follows: the face stimulus and a 9-point Likert scale were both displayed on the screen until the participant responded. To record their answers, participants moved the mouse over the scale and left-clicked at the position that best matched their impression. The next trial started approximately 300 ms later (i.e., a rough estimate of the time it took to compute a stimulus).

#### Analysis and results

**FIGURE 2:**
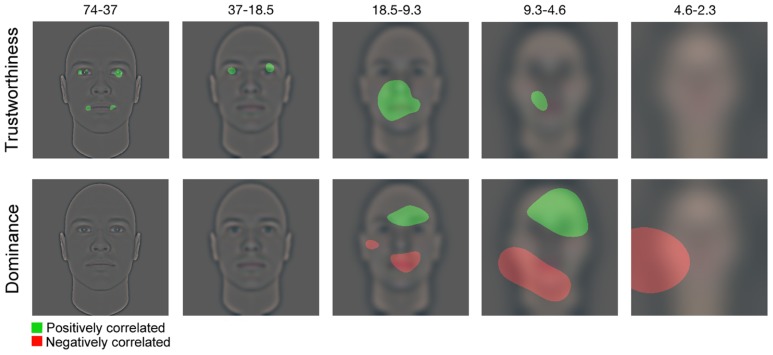
**Significant pixels for each spatial-frequency bandwidth (from fine to coarse) are displayed**.

To uncover the visual features biasing the observers’ social judgment of faces, we performed a least-square multiple linear regression on the Bubbles locations (i.e., pixel locations on which each bubble was centered in each spatial frequency band) and the BIB. To compute the BIB, the following steps were applied: For each participant, (1) we transformed the ratings they gave to the 300 bubblized faces into *Z*-score values, (2) we transformed the ratings they gave to the 300 fully visible faces into *Z*-scores values, and (3) for each identity, we subtracted the *Z*-score rating obtained on the fully visible face (calculated in step 2) from the *Z*-score rating on its bubblized counterpart (calculated in step 1). Thus, the BIB reflects both the direction (positive or negative) and the strength of the bias induced by the presence of bubbles on the stimulus. Once the BIBs were calculated, we computed CIs by calculating a weighted sum of the bubbles’ locations in each trial, using as weight the BIB associated with the face presented. One such CI was produced individually for each participant, spatial frequency band, and judgment type (i.e., trust or dominance). A group CI was then produced for each spatial frequency band and judgment by summing the individual participants’ CIs. We smoothed each grouped CI with the same Gaussian kernels as in the experiment. We then transformed the resulting values into *Z*-scores, using the mean and the standard deviation of the null hypothesis, estimated by repeating the procedure described above with permutations of the BIB. More specifically, the BIB values were randomly shuffled and assigned to the bubbles’ location and a new CI was computed. Then, we estimated the mean and the standard deviation of the null hypothesis in a specific spatial frequency band by calculating the mean and the standard deviation across all the values randomly generated. Note that we repeated these steps five times in order to make sure that the *Z*-scores produced using this procedure were stable. The same results were obtained on every repetition. Finally, we applied the Pixel test to each group CI (*p* < 0.05 two-tailed; Chauvin et al., 2005). **Figure 2** displays, for each spatial frequency band, the facial areas that significantly biased social judgment. The visual information that was significantly positively correlated with the perceived traits (i.e., trustworthiness on the upper row, dominance on the lower row) is depicted in green, and the visual information that was negatively correlated with the perceived traits is depicted in red. The results show that utilizing the eyes in the two highest spatial frequency bands (i.e., between 18.5 and 74 cycles per face), and the mouth in the highest spatial frequency band (i.e., between 37 and 74 cycles per face) as well as in the mid-to-low spatial frequencies (i.e., between 4.6 and 18.5 cycles per face) led to an increase in perceived trustworthiness. No visual information was significantly negatively correlated with perceived trustworthiness. On the other hand, the utilization of the eye/eyebrows area in mid-to-low spatial frequencies (i.e., between 4.6 and 18.5 cycles per face) led to an increase in perceived dominance. The utilization of the mouth area in mid-to-low spatial frequencies (i.e., between 4.6 and 18.5 cycles per face), and the left jaw in low spatial frequencies (i.e., between 2.3 and 9.3 cycles per face) led to a decrease in perceived dominance.

**FIGURE 3:**
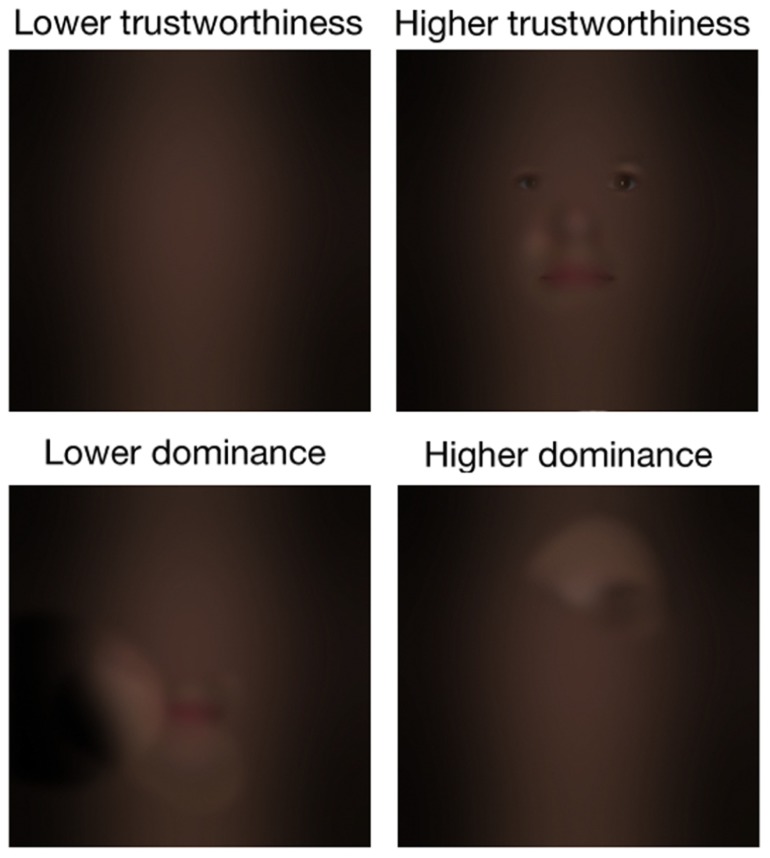
**Classification images that reveal potent information used by participants for each social judgment.** Note that no visual information is significantly correlated with untrustworthiness (only the lowest un-sampled spatial frequency band is represented).

**Figure 3** shows the same base face on which only the visual information significantly correlated with each judgment is made available. Looking at this figure, one can subjectively experience how revealing only a subset of the information contained within a face can alter how trustworthy, dominant or submissive this face looks. However, it is not impossible that these changes in percept were caused as an artifact of the all-or-nothing aspect of the Bubbles. One of the aims of the next experiment was thus to objectively assess how manipulating visual information in a more subtle manner than what is allowed with Bubbles can alter social perception.

## EXPERIMENT 2

During the Bubbles experiment described above, only a small amount of the visual information contained in a face was revealed on each trial (see **Figure 1E**). Thus, one could reasonably ask if the participants processed these bubblized faces using the same perceptual mechanisms as for the fully visible ones. Many researchers believe that face processing is achieved through holistic processing. Moreover, although it has been shown that holistic processing is not *necessary* for performing social judgments (Quadflieg et al., 2012), it has been proposed that, in normal individuals, social judgments are linked to holistic/configural mechanisms (Todorov et al., 2010). A frequent concern regarding the utilization of the Bubbles technique is that it might prevent observers from using holistic processing (Goffaux and Rossion, 2006). One of the aims of Experiment 2 was thus to verify that the results of Experiment 1 generalized to visual conditions where most of the pixels of the stimuli are visible, allowing a global processing of the face. To investigate this, we used the CIs obtained in Experiment 1 to create stimuli in which the variations in the visual information revealed was more subtle than with the dichotomic nature of bubbles. Moreover, as mentioned above, the number of bubbles used in Experiment 1 (50) was somewhat arbitrarily chosen. In Experiment 2, most of the pixels of the face were visible, therefore allowing us to make sure that the results of Experiment 1 were not an artifact of the number of bubbles. Most importantly, we used two different sets of faces (the one used in Experiment 1, as well as another one) to verify whether the results obtained in Experiment 1 with computer-generated faces could be generalized to pictures of real faces.

### METHODS

#### Participants

For the second part of the study, 20 participants who did not take part in Experiment 1 were recruited. All participants were Caucasian and had normal or corrected-to-normal visual acuity.

#### Material and stimuli

We used the same computer-generated faces as in Experiment 1 (Singular Inversions, 2005; Oosterhof and Todorov, 2008) as well as a second set of 276 pictures of Caucasian faces taken from the face database created by Lebrecht et al. (2009). The pictures from Lebrecht et al. (2009) were obtained from the Department of Corrections public face databases of the states of Florida, Arkansas, Georgia and Kansas. Internal facial features were removed from the original pictures and digitally placed into a standard face template, which held hairstyle, clothing, and face contour constant (see details in Lebrecht et al., 2009). The faces from both databases were spatially aligned on the main internal facial features (eyes, mouth, and nose) using translation, rotation and scaling.

**FIGURE 4:**
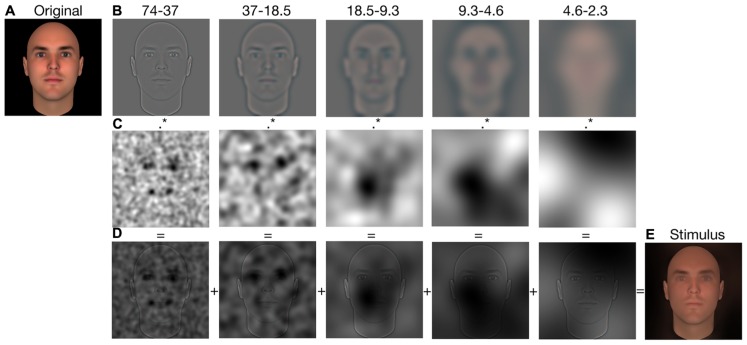
**Illustration of the stimulus-generation process for Experiment 2.** Each original stimulus **(A)** was first decomposed into five spatial-frequency bandwidths **(B)**. Each bandwidth was then independently multiplied by the respective SCI **(C)**. The resulting visual information were summed across the five scales **(D)** to produce a filtered stimulus **(E)**.

**FIGURE 5:**
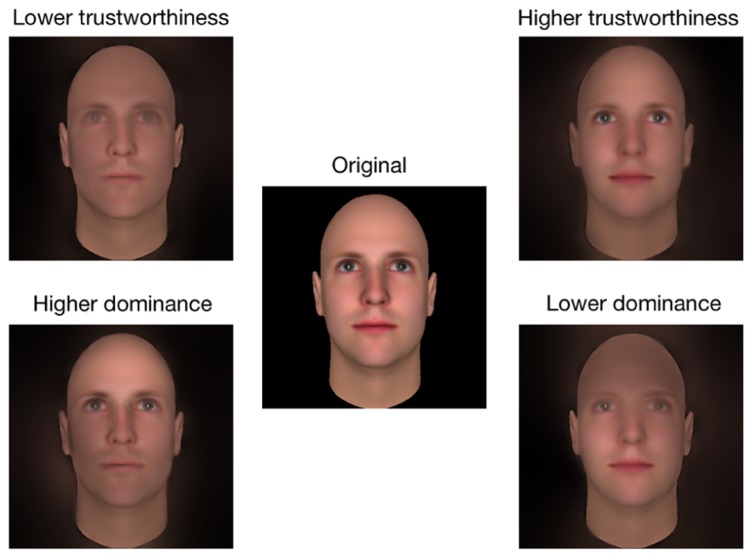
**Examples of the stimuli used in Experiment 2.** Both pro- and anti- version of each social judgment as well as the original stimulus for the computer-generated faces are shown.

**FIGURE 6:**
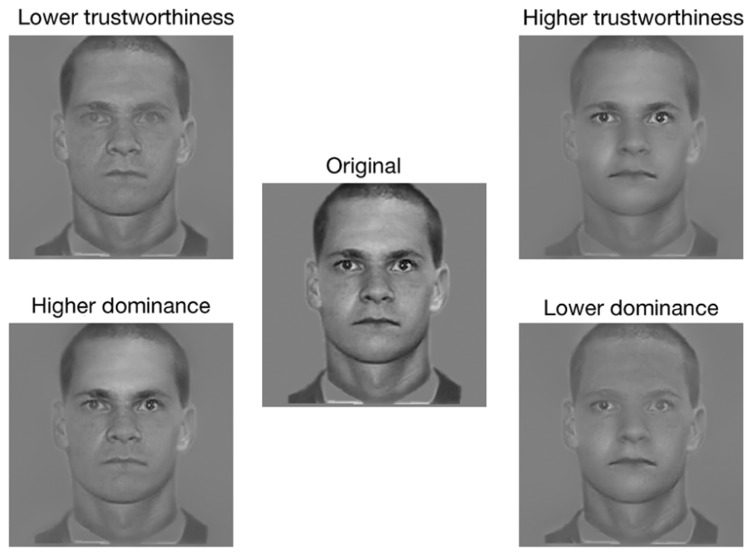
** Examples of the stimuli used in Experiment 2.** Both pro- and anti- version of each social judgment as well as the original stimulus for the natural dataset are shown.

To create an experimental stimulus, the picture of a face (see **Figure 4A**) was first bandpass filtered into five non-overlapping spatial frequency bands (74–37, 37–18.5, 18.5–9.3, 9.3–4.6, 4.6–2.3 cycles per face; the remaining bandwidth served as a constant background; see **Figure 4B**), using the Pyramid toolbox for Matlab (Simoncelli, 1999). Then, we scaled the values contained in the raw CIs (i.e., before a significance threshold was applied) such that the minimum value became 0 and the maximum value became 1 (we will henceforth refer to these new CIs as the Scaled CIs (SCIs); see **Figure 4C**). Thus, the relative distance between the values contained in the SCIs remained the same as for the CIs. Areas in the SCIs that were highly positively correlated with a judgment had a value of 1, whereas those that were highly negatively correlated with a judgment had a value of 0; other values were somewhere in-between. To create a face in which the visual information leading to an increase of the perceived trustworthiness was emphasized, we dot-multiplied the SCI for trustworthiness by a base face. In contrast, to create a face in which the visual information leading to a decrease of the perceived trustworthiness was emphasized, we dot-multiplied the inverse of SCI for trustworthiness by a base face (thus decreasing the visibility of the visual information that biased the judgment toward higher trustworthiness; e.g., see **Figures 4D,E**). The same procedure was used to create faces in which the visual information leading to an increase (or a decrease) of the perceived dominance was emphasized (or diminished). We applied this procedure on each face of both databases. This allowed us to obtain a pro- and an anti-version of each social judgment for each original stimulus. The unfiltered versions of the faces were also presented as a baseline measurement. **Figures 5** and **6** display examples of faces (i.e., one face from the computer-generated database and the other one from the Lebrecht et al., 2009 database) revealed through the pro- and anti-filter of each judgment.

The stimuli were displayed on two 21.5-inch iMac computers with a refresh rate of 60 Hz. The experimental program was written in Matlab, using functions of the Psychophysics Toolbox (Brainard, 1997; Pelli, 1997).

#### Procedures

Four tasks each containing 150 trials were conducted. Tasks 1 and 2 included the computer-generated faces, and tasks 3 and 4 included the real face pictures. In tasks 1 and 3, the participants were asked to make trustworthiness judgments whereas in tasks 2 and 4, they were asked to make dominance judgments. The order in which the four tasks were conducted was counterbalanced. In each task, 50 faces were presented under three different conditions: filtered with the visual information increasing a percept, filtered with the visual information increasing the opposite percept, or not filtered at all. The order of the faces and the conditions were randomly selected. On each trial, the participants were asked to judge a face on its level of trustworthiness or dominance on a scale that ranged from 1 (*not trustworthy* or *not dominant* at all) to 9 (*very trustworthy* or *very dominant*). Judgments obtained for fully visible faces served as the baseline, while judgments obtained from the filtered versions of the same faces were assumed to reflect the influence that our subtle luminance variations had on the percept.

#### Analysis and results

**FIGURE 7:**
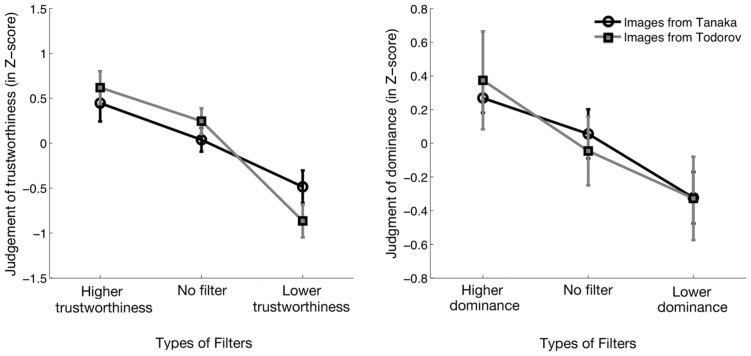
**Average normalized judgments according to different conditions of filtering (pro-[trait], neutral, anti-[trait]) in function of the two databases of faces**.

For each of the four tasks, the participants performed 150 trials (50 pro-, 50 neutral, and 50 anti-). In each task, the ratings of each participant were transformed into *Z*-score values, using the mean and the standard deviation of the ratings across the 150 trials. The *Z*-scores were submitted to a repeated measures ANOVA on the factor of the type of filter (i.e., pro, neutral, or anti) through which the face was revealed (see **Figure 7**). The effect of the type of filter was significant in all four experiments (Task 1: *F*(2,38) = 273.4, *p* < 0.001, η^2^ = 0.94; Task 2: *F*(2,38) = 26.6, *p* < 0.001, η^2^ = 0.58; Task 3: *F*(2,38) = 94.8 *p* < 0.001, η^2^ = 0.83; Task 4: *F*(2,38) = 68.5, *p* < 0.001, η^2^ = 0.78). We then conducted paired-samples *t*-tests to investigate if the pro-filters led to an increase in the perceived trustworthiness (i.e., Tasks 1 and 3) or dominance (i.e., Tasks 2 and 4), compared to the baseline judgments, and if the anti-filters led to a decrease in the perceived trustworthiness or dominance, compared to the baseline judgments. Bonferroni corrections were applied within each task to correct for the multiple comparisons performed. For trustworthiness judgments, the pro-filter led to a significant increase in perceived trustworthiness for both face databases when compared with the neutral filter (Computer-generated: *t*(19) = 6.1, *p *< 0.001; Real face pictures: *t*(19) = 6.3, *p* < 0.001). The anti-filter had the opposite effect, leading to a significant decrease of perceived trustworthiness for both face databases (Computer-generated: *t*(19) = 18.2, *p* < 0.001; Real face pictures: *t*(19) = 9.6, *p* < 0.001). Filters had the same effect on dominance judgments as what was observed in the trustworthiness task, i.e., the pro-filter led to a significant increase in perceived dominance (Computer-generated: *t*(19) = 4.3, *p* < 0.001; Real face pictures: *t*(19) = 5.1, *p* < 0.001), and the anti-filter led to a significant decrease in perceived dominance (Computer-generated: *t*(19) = 3.6, *p* < 0.005; Real face pictures: *t*(19) = 5.9, *p* < 0.001), when compared to the neutral filter. Thus, our results indicate that it is possible to manipulate social judgments by subtly manipulating the visibility of the visual information contained in a face. They also show that the CIs revealed in Experiment 1 are not specific to the face database used, nor to the number of bubbles chosen, and that they generalize to another database comprising pictures of real faces.

## DISCUSSION

The present study investigated the visual information subtending social judgments for faces at both the featural and spatial frequency levels. In Experiment 1, the Bubbles method was used to reveal the visual information biasing facial judgments toward higher (vs. lower) trustworthiness and toward higher (vs. lower) dominance. In Experiment 2, we investigated whether we could manipulate the perceived trustworthiness or dominance of a face by using the CIs obtained in Experiment 1 to apply subtle changes to the relative contrast across the pixels of the image on two different face databases.

### THE VISUAL INFORMATION UNDERLYING TRUSTWORTHINESS AND DOMINANCE JUDGMENTS

The results of Experiment 1 showed that the use of the eye and the mouth areas was positively correlated with trustworthiness judgments. More specifically, the perceived trustworthiness of a face increased (vs. decreased) when the eye area and the corners of the mouth were revealed (vs. not revealed) in high spatial frequency, and/or when the mouth area was revealed (vs. not revealed) in medium to medium-low spatial frequency bands. Alternatively, no specific region was negatively correlated with perceived trustworthiness. In other words, there were no areas that, when revealed, decreased the perceived trustworthiness. Moreover, perceived dominance was positively correlated with the utilization of the eyebrows’ region in the medium-low to medium spatial frequency bands. That is, when that visual information was revealed, perceived dominance increased, whereas when it was not revealed, perceived dominance decreased. The percept of dominance was also negatively correlated with utilization of the mouth area in mid-low spatial frequencies and of the left jaw in low spatial frequencies, such that a face was perceived as less dominant when that information was revealed, and as more dominant when it was not revealed.

The spatial part of the results obtained in Experiment 1 is congruent with previous studies that have looked at the link between facial appearance and trustworthiness or dominance judgments (Oosterhof and Todorov, 2008; Dotsch and Todorov, 2012). In fact, similarly to these studies, we show that the eye/eyebrows, the mouth, and the jaw areas are correlated with the percept of trustworthiness and dominance. Furthermore, we show that spatial coordinates and spatial frequencies interact to modulate the two judgments. For instance, while the eye/eyebrows area must be revealed in high-spatial frequencies to modulate the trustworthiness percept, it must instead be revealed in medium-low spatial frequencies to modulate the dominance percept. To our knowledge, this nuance is a completely new addition to the literature. Most importantly, it is this new piece of information that has allowed us to manipulate the stimuli presented in Experiment 2 on both their spatial and spectral domains’ relative contrasts, thereby producing stimuli that appear more natural (vs. Bubblized ones).

Previous studies have shown that low levels of trustworthiness (Oosterhof and Todorov, 2008; Dotsch and Todorov, 2012) or high levels of dominance (Hess et al., 2000; Montepare and Dobish, 2003) are associated with the presence of facial features found in the facial expression of anger. In light of this, one could expect to find an overlap between the information utilization pattern biasing judgments toward lower levels of trustworthiness or higher levels of dominance, and the information utilization pattern for anger recognition. It has been shown that recognition of the facial expression of anger relies on the eye and the eyebrow areas (Smith et al., 2005). The results obtained in Experiment 1 for dominance judgments, i.e., an increase in the perceived level of dominance following the utilization of the eye/eyebrows’ region in the medium-low to medium spatial frequency bands, are consistent with the visual information found to be useful for anger recognition. However, the positive (rather than negative) correlation between the eye-eyebrows area and the percept of trustworthiness in our data is intriguing. One possible explanation is that someone attempting to judge the trustworthiness of a face might attempt to use the eye region to check for anger-like information: If they find that the features do not conform with it, then trust increases. In contrast, if they find that the feature conforms with the anger expression, then trust decreases. Because we used neutral expression faces, there could have been little in the way of giving off cues of anger. Since the eye area of our stimuli did not reveal anger cues, such a strategy might most often have led to an increase (rather than a decrease) of the perceived trustworthiness, thereby leading to the positive correlation observed. Another possibility is that when the eyes were revealed, the participants’ attention was automatically drawn toward that feature, and this induced an impression of sustained eye-contact. In fact, it is often assumed in folk psychology that trustworthy people keep eye-contact, whereas untrustworthy people do not. Of course, these two possible explanations remain speculative, and more research will be needed to better understand the origin of the correlation between eye utilization and perceived trustworthiness.

### HOW TO RIG SOCIAL JUDGMENTS

**FIGURE 8:**
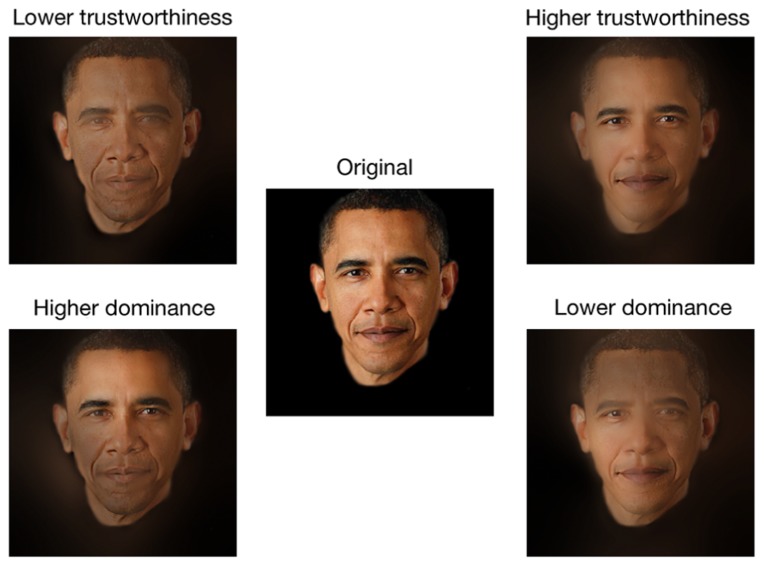
**As a final exploration of the generalizability of our manipulation, we set out to find one of the most emblematic figures on earth.** Google spoke: Barack Obama. The intact image (center) was passed through an untrustworthy (top left), trustworthy (top right) dominant (bottom left), and a submissive filter (bottom right). Original image source: http://change.gov/newsroom/entry/new_official_portrait_released/. Photo licensed under the Creative Commons Attribution 3.0 License (http://creativecommons.org/licenses/by/3.0/).

One of the main differences between the Bubbles method and the methods used in preceding studies is that the former does not alter the features’ shape, but instead either hides or reveals them. Thus, the results of Experiment 1 allow to conclude that it is possible to rig trustworthiness and dominance perception without altering the features’ shape by instead modulating the relative saliency of different facial areas. However, one drawback of Experiment 1 was that only a small amount of the visual information contained in a face stimulus was available on each trial, and these incomplete stimuli may have been processed with atypical strategies. Thus, in Experiment 2, we showed that the raw CIs (i.e., before a statistical threshold was applied) obtained in Experiment 1 could be used to subtly modulate the relative contrast of the visual information contained in a face, and that this manipulation powerfully and reliably rigged perceived dominance and trustworthiness. It is important to note that the manipulations applied on the stimuli of Experiment 2 may have slightly changed the global shape of the face and/or the global shape of some features, but these changes were very subtle. For instance, the identity of the face remained unchanged and easily recognizable (for an example of the manipulation applied on a familiar face, see **Figure 8**), and the facial expression did not drastically change from one of anger to one of happiness. From our point of view, **Figure 8** is a strong indicator that the contrast manipulation performed in the present study might even generalize to well known faces.

Another difference between the Bubbles method and the methods used in the preceding studies is that the former allowed us to investigate in which spatial frequencies the different facial areas were used when the trustworthiness or dominance percept was modulated. One unexpected advantage of the spatial frequency sampling in Experiment 1 is that the application of the resulting raw CIs on base faces in Experiment 2 modulated global features such as the skin texture. For instance, a comparison of the lower trustworthiness and higher trustworthiness exemplars displayed on **Figure 6** (see also **Figure 8**) reveals that while the skin of the higher trustworthiness exemplar appears to be smooth and healthy, the one of the lower trustworthiness exemplar appears imperfect. This is congruent with recent evidence that faces with healthy skin are perceived as more trustworthy (Dzhelyova et al., 2013). Furthermore, in Experiment 2, we showed that the CIs produced in Experiment 1 can be applied to a completely different set of faces and still successfully modulate perception. Thus, our results suggest that most faces contain visual information linked with trustworthiness *and* untrustworthiness, as well as information linked with dominance *and* submission. By highlighting some features at the expense of others, it is therefore possible to modulate the percept. In light of this discussion, the practical implications of these result could become quite attractive to fields that employ image branding, such as political campaigns, marketing, and anchoring. For example, our results could pave the way to a more formal theoretical background to the tradition of applying makeup (Scherbaum et al., 2011) to public figures.

## CONCLUSION

To summarize, we showed what visual information underlies the fluctuations observed in two types of social judgments, and that this knowledge can be used to manipulate facial appearance and rig social perception. Further research will be needed to better understand why the utilization of the visual information revealed in this study influences social perception, as well as how this information utilization is linked to the activity of brain structures involved in the social perception of faces. Nonetheless, our results open the way to an interesting and powerful tool for manipulation of facial appearance. Such a tool could, for instance, be used during electoral campaigns, to ever-so-subtly modify both the image of your candidate through a pro-attribute filter, *and* that of your opponent through an anti-attribute filter.

## Conflict of Interest Statement

The authors declare that the research was conducted in the absence of any commercial or financial relationships that could be construed as a potential conflict of interest.
